# Development of a Single Vector System that Enhances *Trans*-Splicing of *SMN2* Transcripts

**DOI:** 10.1371/journal.pone.0003468

**Published:** 2008-10-22

**Authors:** Tristan H. Coady, Travis D. Baughan, Monir Shababi, Marco A. Passini, Christian L. Lorson

**Affiliations:** 1 Department of Veterinary Pathobiology, Bond Life Sciences Center, University of Missouri, Columbia, Missouri, United States of America; 2 Department of Molecular Microbiology and Immunology, Bond Life Sciences Center, University of Missouri, Columbia, Missouri, United States of America; 3 Neuroscience, Genzyme Corporation, Framingham, Massachusetts, United States of America; Centre de Regulació Genòmica, Spain

## Abstract

RNA modalities are developing as a powerful means to re-direct pathogenic pre-mRNA splicing events. Improving the efficiency of these molecules *in vivo* is critical as they move towards clinical applications. Spinal muscular atrophy (SMA) is caused by loss of *SMN1*. A nearly identical copy gene called *SMN2* produces low levels of functional protein due to alternative splicing. We previously reported a *trans*-splicing RNA (tsRNA) that re-directed *SMN2* splicing. Now we show that reducing the competition between endogenous splices sites enhanced the efficiency of *trans*-splicing. A single vector system was developed that expressed the *SMN* tsRNA and a splice-site blocking antisense (ASO-tsRNA). The ASO-tsRNA vector significantly elevated SMN levels in primary SMA patient fibroblasts, within the central nervous system of SMA mice and increased SMN-dependent *in vitro* snRNP assembly. These results demonstrate that the ASO-tsRNA strategy provides insight into the *trans*-splicing mechanism and a means of significantly enhancing *trans*-splicing activity *in vivo*.

## Introduction

*Trans*-splicing has recently been envisioned as a potential therapeutic intervention for a variety of genetic diseases. The potential effectiveness of this strategy has been demonstrated in a variety of diseases including spinal muscular atrophy, cystic fibrosis, hyper-IgM X-linked immunodeficiency, hemophilia A, Alzheimer's disease, and epidermolysis bullosa simplex with muscular dystrophy [1]–[6]. *Trans*-splicing is a natural, albeit infrequently utilized process in mammals, therefore, maximizing efficiency is central to developing *trans*-splicing therapeutics [7]. Conceptually, this strategy relies upon nuclear pre-mRNA splicing occurring between two different molecules: 1) the mutant endogenous RNA and 2) the exogenous therapeutic RNA that provides the correct RNA sequence via a *trans*-splicing event.

Spinal Muscular Atrophy (SMA), a neurodegenerative disorder, is caused by the homozygous loss of *survival motor neuron 1* (*SMN1*) and is the leading genetic cause of infantile death [8], [9]. In humans two copies of the *SMN* gene exist, *SMN1* and *SMN2* [10]. The critical distinction between the two genes occurs at the RNA processing level: *SMN1* produces full-length transcripts, while *SMN2* primarily produces an alternatively spliced transcript lacking the final coding exon [10]. A single silent C to T non-polymorphic nucleotide difference is responsible for disrupting a critical splice enhancer element in *SMN2* exon 7 [11], [12].

*SMN2* is retained in essentially all SMA patients and is a primary target for SMA therapeutic development [13]. In addition to the identification and development of small molecules that stimulate full-length *SMN2* expression, RNA modalities such as antisense oligonucleotides (ASO), TOES/bifunctional RNAs and *trans*-splicing RNAs have shown promise in SMA cell-based models [14]–[20]. ASOs have also been shown to modulate *SMN2* expression *in vivo* in an unaffected transgenic mouse expressing the human *SMN2* gene [18].

In this report, we demonstrate that *trans*-splicing efficiency is enhanced by competitively disabling a downstream splice site. As a means to develop a tractable molecular modality, a series of antisense RNAs were screened to identify a sequence that would disable *SMN* exon 8 and promote *trans*-splicing. *In vitro* assays identified an enhancing antisense RNA and were constructed into a novel single vector system individually expressing the *trans*-splicing RNA and the antisense RNA. Cell-based assays identified a highly efficient vector system that resulted in high levels of trans-splicing and correspondingly high SMN protein levels and increased SMN activity in SMA-derived extracts as measured by snRNP assembly assays. Intracerebroventricular delivery of the ASO-tsRNA vector in the SMA mouse model increases SMN protein in the central nervous system of affected animals, demonstrating a platform that can significantly elevate SMN levels *in vivo* and in a relevant disease context.

## Results

### Competitive inhibition of downstream splice site enhances *SMN trans*-splicing

The key to introducing *trans*-splicing *in vivo* and in clinical settings is by developing efficient *trans*-splicing systems. We have previously described a *SMN trans*-splicing system that significantly elevated full-length SMN protein levels in SMA patient fibroblasts [15]. However, initial *in vivo* experiments failed to achieve similar levels of activation (data not shown). Therefore, we initially examined SMN2 trans-splicing with the goal of devising strategies to enhance trans-splicing efficiency *in vivo*.

**Figure 1:**
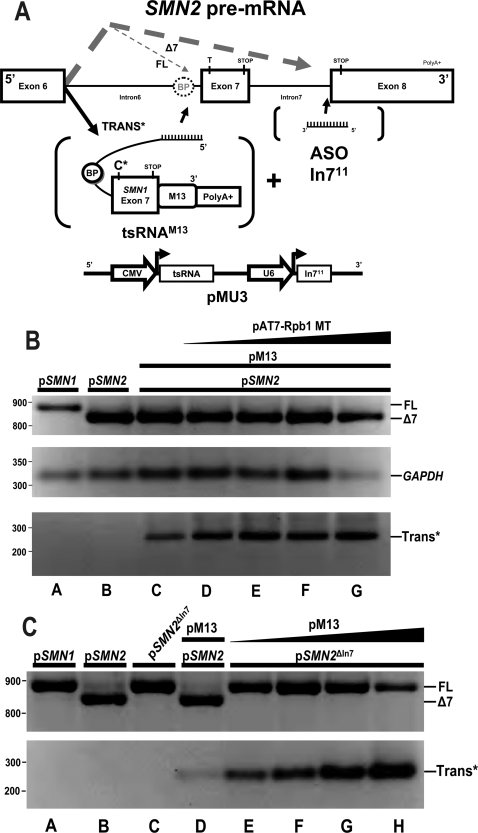
Development of a single plasmid vector enhances *SMN* based *trans*-splicing. (A) Proposed ASO-tsRNA mechanism. *SMN2* transcripts alternative splice producing two mRNA products. The alternative splicing pathway is represented by gray dashed lines, *SMN2* mRNA products: “Δ7”- Exon 7 skipped, “FL”- full length. *Trans*-splicing RNA identifies *SMN2* pre-mRNA intron 6 which overlaps the endogenous branch point then incorporated in the final mRNA is catalyzed by the spliceosome. Circles surrounding symbol “BP” identifies known branchpoints in the model. We demonstrate a novel mechanism of disrupting downstream intron 7 splicing elements causes enhancement of *trans*-splicing. (Bold “TRANS*” and large black arrow indicate enhanced *trans*-splicing pathway). The effect is accomplished via enhancing Antisense Oligonucleotides “ASO In7^11^” targeted to the distal intron and exon boundary. The stop codons “STOP” and *SMN2* “C-T” or tsRNA “C*” nucleotide changes are denoted with vertical line marking approximate RNA position. Bracketed objects indicate promoters and gene products produced from pMU3 plasmid pictured below by a black line. (B) Increasing concentrations of transcription mutant RNA polymerase promotes *trans*-splicing. HeLa cells were transfected of static amounts mini-gene p*SMN1* (lane a) or p*SMN2* (lanes b–g) 1.25 µg , pM13 (lanes c–g) 0.75 µg, and increasing amounts of mutant Rpb1 RNA Polymerase subunit (pAT7-Rpb1MT) at 0.25, 0.75, 1.0 and 2.0 µg were harvested at 48 hrs. Reverse transcriptase PCR gel is displayed with *GAPDH* normalization control. (C) A genetically disabled SMN2 intron 7 deletion mini-gene displays enhances trans-splicing at reduced tsRNA plasmid concentrations. HeLa cells were cotransfected with p*SMN1* (lane a), p*SMN2* (lanes b,d), p*SMN2* ^ΔIn7^ (lanes e–h) 1.25 µg each and increasing concentrations of pM13 (lane d) 0.25 µg, (lanes e–h) 0.10, 0.25, 1.0 and 2.0 µg and the RNA harvested at 48 hrs. Reverse transcriptase PCR gel is displayed with *GAPDH* normalization control.

In *SMN2 trans*-splicing, there are three potential splicing outcomes: full length (FL), truncated (Δ7) and *trans*-spliced mRNA (TRANS*) (Figure 1A). Since pre-mRNA splicing is a highly dynamic process, we hypothesized that competition exists between the *trans*-splicing event and the naturally strong *SMN2* exon 8 splice site. Thus, competitive inhibition of the exon 8 splice site would enhance *trans*-splicing efficiency. To address this possibility, two complementary experiments were performed. A previously described slow-polymerase encoding plasmid was co-transfected with the *SMN2* mini-gene and the plasmid expressing the *SMN trans*-splicing RNA [21]. Under these experimental conditions, the exon 8 3′ splice site approximately two kb downstream from exon 7, should not be synthesized as quickly as the natural polymerase, therefore, potentially favoring the *trans*-splicing event. Consistent with the hypothesis, in the presence of the slow transcribing polymerase, *trans*-splicing was substantially increased over the typical level of *trans*-splicing previously observed (Figure 1B). Additionally, a *SMN2* mini-gene was used in a separate experiment in which the intervening intron between *SMN2* exons 7 and 8 was deleted. In the absence of an intact acceptor site at the intron 7/exon 8 junction, *SMN trans*-splicing was again elevated several fold over the standard *SMN2* mini-gene (Figure 1C). Collectively, these results demonstrate that competitively disabling the downstream splice site can lead to enhanced *trans*-splicing efficiency, either through a biochemical means or by a genetic alteration.

### Identification of anti-sense RNA that enhances *SMN trans*-splicing

**Figure 2:**
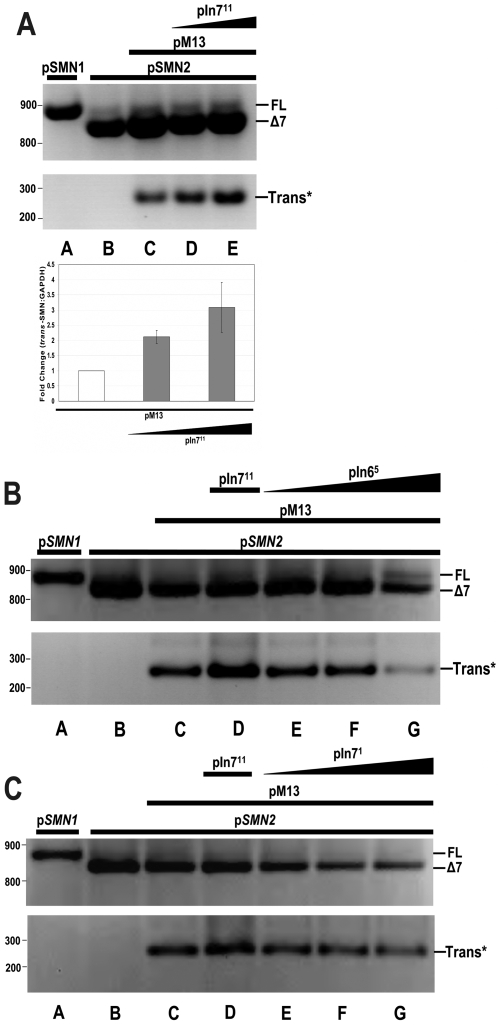
ASO mediated inhibition of downstream splicing increases *SMN2 trans*-splicing. (A) HeLa cells transfected with static amounts of p*SMN1* (lane a), p*SMN2* 1.25 µg (lanes b–e) and pM13 (lane c–e) 0.75 µg are cotransfected with increasing concentrations of pIn7^11^ (lane d,e) 0.25, 1.0 µg and RNA harvested at 48 hrs. Reverse transcriptase PCR gel is displayed with *GAPDH* normalization control. Graph represents independent triplicate repeats using a quantitative M13-Cy3 labeled primer and linear-range PCR amplification. Inset graph represents the average of triplicate repeats and error bars indicate ±s.d. (B) *SMN* intron 6 ASO controls contribute to a reduction of *trans*-splicing. HeLa cells were triple transfected with static amounts of mini-gene p*SMN1* (lane a) p*SMN2* (lane b–g) 1.25 µg and pM13 (lane c–g) 0.75 µg with increasing amounts of pIn6^5^ (lane e–g) 0.25, 0.75 and 1.0 µg and RNA harvested at 48 hrs. Reverse transcriptase PCR gel is displayed with *GAPDH* normalization control. pIn7^11^ (lane c) 1.0 µg serves as a positive ASO-tsRNA control. (C) ASOs targeted internally to *SMN* intron 7 reduce *trans*-splicing. HeLa cells were triple transfected with static amounts of mini-gene p*SMN1* (lane a) p*SMN2* (lanes b–g) 1.25 µg and pM13 (lanes c–g) 1.0 µg with increasing amounts of pIn7^1^ (lanes e–g) 0.25, 0.75 and 1.0 µg and RNA harvested at 48 hrs. Reverse transcriptase PCR gel is displayed with *GAPDH* normalization control. pIn7^11^ (lane c) at 0.25 µg serves as a positive ASO-tsRNA control.

The previous experiments provided the impetus to identify a more tractable molecular mechanism to enhance *SMN trans*-splicing, such as antisense molecules. Antisense oligonucleotides (ASO) have proven to be an effective molecular means to modulate pre-mRNA splicing, primarily by inhibiting splice site selection. A panel of plasmids expressing short 18–22 nt ASOs complementary to the intron 7/exon 8 splice site were constructed and used to determine whether any of the *SMN* antisense RNAs could enhance *trans*-splicing. (Supplemental Figure S1A, S1B, see Methods) The ASO-expressing plasmids were co-transfected into HeLa cells with the *SMN2* mini-gene and the pM13 which expresses the *SMN trans*-splicing RNA. A single lead candidate was identified that significantly elevated *trans*-splicing levels: ASO pIn7^11^. ASO In7^11^ overlaps the intron 7/exon 8 boundary (Figure 2A). Other ASO did not increase *trans*-splicing from the *SMN2* mini-gene, or in some instances, even inhibited *trans*-splicing (Figure 2B, 2C). These results confirm the genetic experiments that ASO inhibition of a downstream splice site can significantly enhance *trans*-splicing efficiency in a mini-gene context.

**Figure 3:**
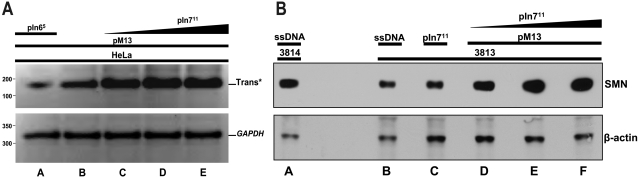
Endogenous enhancement of *trans*-splicing utilizing combined delivery of ASO and tsRNA. (A) Dose dependant ASO In7^11^ enhancement of endogenous *SMN trans*-splicing. HeLas were cotransfected with pM13 (lanes a–e) 1.0 µg and increasing concentrations of ASO pIn7^11^ (lanes c–e) 0.25, 2.0 and 5.0 µg and RNA harvested at 48 hrs. Reverse transcriptase PCR gel is displayed with *GAPDH* normalization control. pIn6^5^ (lane a) 2.0 µg serves as a negative control. (B) Dose dependant ASO pIn7^11^ enhancement of pM13 mediated SMN protein induction. 3813 SMA patient fibroblasts were co-transfected with pM13 (lanes d–f) 0.75 µg and increasing concentrations of pIn7^11^ (lanes d–f) 0.50, 1.0 and 2.0 µg and cells harvested at 24 hrs and run on 10% SDS-PAGE. pIn7^11^ alone (lane c) 2.75 µg and ssDNA (lanes a, b) 2.75 µg serve as negative controls. β-actin antibody panel serves as a normalization control.

To determine whether ASO-enhanced *trans*-splicing occurred in a more complex context of endogenous *SMN* gene expression, pIn7^11^ and pM13 was co-transfected into HeLa cells to examine *trans*-splicing with endogenous *SMN* transcripts. HeLa cells express *SMN1* and *SMN2* genes and provide a robust level of target RNA. Consistent with the mini-gene analysis, endogenously derived *trans*-spliced *SMN* increased in a dose dependant manner with increasing amounts of pIn7^11^ (Figure 3A). To examine *trans*-splicing in a more disease-specific context, similar co-transfections were performed in primary SMA patient fibroblasts, 3813 cells. The cells are derived from a severe Type I SMA patient and lack endogenous *SMN1*, and consequently contain very low levels of SMN-enriched nuclear structures called gems and express very low levels of full-length SMN protein. Extracts generated from 3813 cells co-transfected with pM13 and increasing concentrations of ASO pIn7^11^ were analyzed for SMN levels using a SMN monoclonal antibody. Western blots demonstrated an ASO dose-dependent protein induction greater than control-treated 3813 cells as well as pIn7^11^ alone treated cells (Figure 3B). SMN levels in the co-transfected cells were comparable to SMN levels detected in the unaffected control fibroblasts, 3814 cells. These results demonstrate that endogenous *SMN* transcripts can be re-directed by *trans*-splicing and that SMN levels can be significantly increased by the ASO/*trans*-splicing strategy in a relevant disease context.

### Development of a single vector for anti-sense and *trans*-splicing RNA delivery

**Figure 4:**
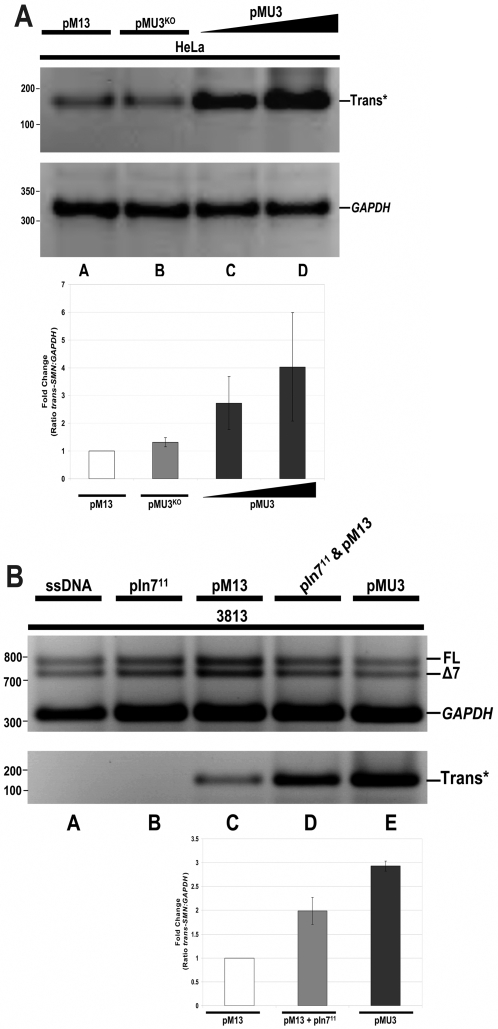
Development of a single plasmid ASO-tsRNA system (pMU3) enhances endogenous *SMN trans*-splicing. (A) pMU3 enhances *trans*-splicing capabilities in a single vector. HeLas were transfected with pM13 (lane a) 0.75 µg or pMU3^KO^ (lane b) 0.75 µg or pMU3 (lanes c,d) 0.75 and 1.0 µg and RNA harvested 48 hrs later. Reverse transcriptase PCR gel is displayed with *GAPDH* normalization control. Inset graph represents triplicate repeats. (B) In SMA relevant contexts the single plasmid ASO-tsRNA system (pMU3) produces the greatest amount of *trans*-splicing. 3813 SMA patient fibroblasts were transfected with pM13 for basal level (lane c) 0.75 µg or cotransfected with pIn7^11^ 0.50 µg and pM13 0.50 µg together (lane d) or pMU3 (lane e) 0.75 µg and RNA harvested at 48 hrs. ssDNA (lane a) 0.75 µg and pIn7^11^ alone (lane b) 2.0 µg serve as negative controls. Reverse transcriptase PCR gel is displayed with *GAPDH* normalization control. Inset graph represents the average of triplicate repeats and error bars indicate ±s.d.

In envisioning a therapeutic application, a dual vector strategy is likely to reduce the efficiency of *trans*-splicing. Therefore, a single vector was constructed that was combined the molecular constituents of pM13 and pIn7^11^ by subcloning the *SMN trans*-splicing cassette into the pIn7^11^ plasmid backbone. This single vector platform allows for the production of two individual RNAs that target distinct aspects of *SMN* pre-mRNA, leading to enhanced full-length SMN expression (Figure 1A). The ASO-tsRNA plasmid (pMU3) produces three gene products, the Pol II-derived tsRNA^M13^, the Pol III-derived ASO In7^11^, and the Pol II-derived eGFP. Consistent with the co-transfections, the single pMU3 vector resulted in high levels of *SMN trans*-splicing between endogenous HeLa cell *SMN* transcripts. *SMN* levels were elevated nearly three fold compared to the pM13 vector and a pMU3 vector that lacks the promoter that drives the In7^11^ RNA (pMU3^KO^) (Figure 4A). The pMU3 vector was also capable of re-directing endogenous *SMN* pre-mRNA splicing in primary SMA fibroblasts, resulting in approximately a three-fold increase over pM13 levels of *trans*-splicing and a comparable increase in steady state levels of SMN protein (Figure 4B).

### *Trans*-splicing restores SMN-dependent major and minor snRNP assembly

**Figure 5:**
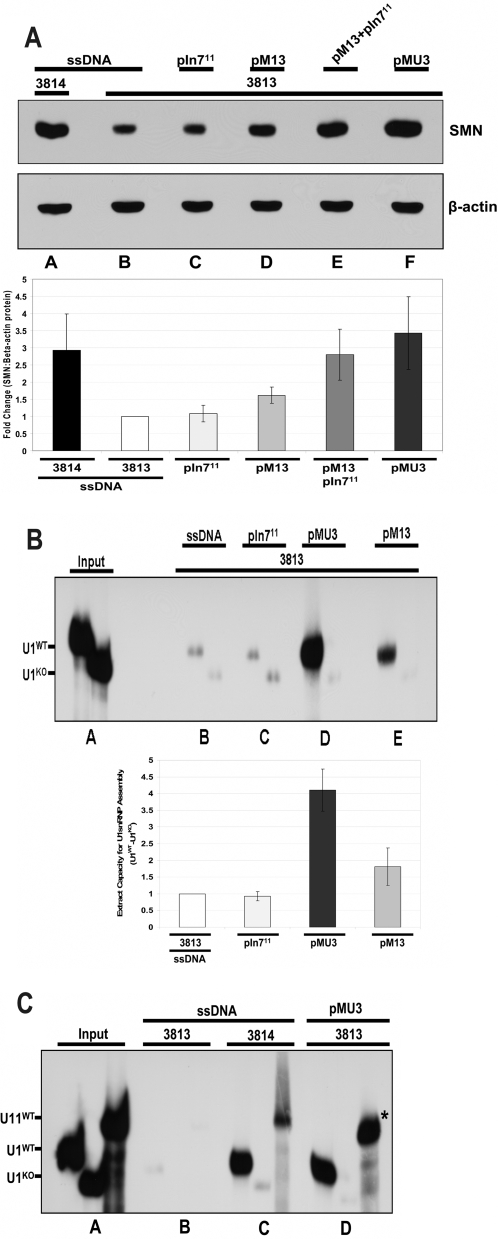
Single vector dosages with enhanced *trans*-splicing produces functional SMN protein in patient fibroblasts. (A) The pMU3 single plasmid ASO-tsRNA system produces the greatest amount SMN protein induction. 3813 SMA patient fibroblasts were transfected with pM13 for basal level (lane d) 0.75 µg or cotransfected with pIn7^11^ 0.50 µg and pM13 0.50 µg together (lane e) or pMU3 (lane f) 0.75 µg cells were harvested at 24 hrs and run on 10% SDS-PAGE. ssDNA (lanes a,b) 0.75 µg and pIn7^11^ alone (lane c) 2.0 µg serve as negative controls. β-actin antibody panel serves as a normalization control. Inset graph represents the average of triplicate repeats and error bars indicate ±s.d. (B) The pMU3 system restores SMA primary fibroblasts *in vitro* capacity to assemble U1snRNP. 3813 SMA patient fibroblasts were transfected with pM13 (lane e) 0.75 µg for basal level or pMU3 (lane d) 0.75 µg cells were harvested at 24 hrs and S100 extract prepared. ssDNA (lane b) 0.75 µg and pIn7^11^ alone (lane c) 2.0 µg serve as negative controls. Inset graph represents the average of triplicate repeats and error bars indicate ±s.d. (C) The pMU3 system restores the SMA primary fibroblasts *in vitro* capacity for minor spliceosome U11 snRNP assembly. 3813 SMA patient fibroblasts were transfected with pMU3 (lane d) 0.75 µg or ssDNA (lanes b,c) 0.75 µg. ssDNA. 3813 and 3814 (lanes b and c) serve as negative and positive controls respectively for U1 and U11^ATAC^ assembly. (*) indicates band of interest.

To monitor the functionality of the SMN protein produced downstream of the *trans*-splicing event, SMN protein function was measured by *in vitro* snRNP assembly assays. The best described activity for SMN is a role in UsnRNP assembly [22]. Using SMN-deficient extracts from 3813 cells that normally support very low levels of snRNP assembly, extracts were generated from cells transfected with salmon sperm DNA, pM13, pIn7^11^, or pMU3. Extracts derived from pMU3 transfected cells exhibited a two to four-fold increase compared to untreated extracts, demonstrating that the *trans*-splicing event not only generates higher levels of protein, but that SMN protein produced through this pathway is functional in a critical cellular process ascribed to native SMN (Figure 5A, 5B). These assays examined the ability of SMN to form snRNP on a U1 snRNA, which is part of the major spliceosomal pathway, however, SMN has also recently been shown to be involved in snRNP biogenesis of the minor spliceosome pathway, including the U11^ATAC^snRNA [23]. To determine if this critical function was also restored in *trans*-splicing treated cell extracts, similar *in vitro* assembly assays were performed using a U11^ATAC^snRNA (Figure 5C). Consistent with the U1 results, pMU3 treated extracts were able to significantly elevate functional SMN levels as measured by U11^ATAC^snRNP assembly. Collectively, these results demonstrate that the pMU3 system results in high levels of functional SMN protein that is capable of performing UsnRNP biogenesis of the major and minor spliceosomal pathways to levels comparable to unaffected cells.

**Figure 6:**
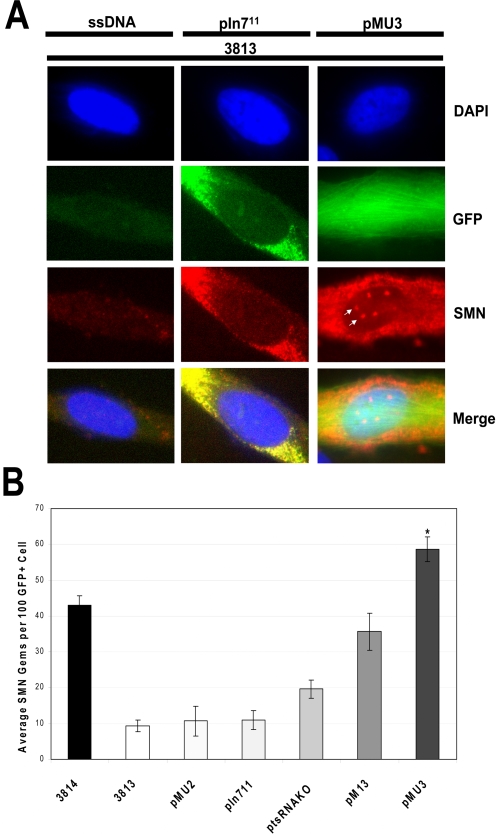
pMU3 transfected SMA primary fibroblasts display increased gem numbers. (A) 3813 SMA patient fibroblasts were transfected with pMU3 0.75 µg or ssDNA 0.75 µg and fixed at 24 hrs. Immunohistochemistry was performed using αSMN antibody 4B7 and visualized with Texas Red 594, nuclei with DAPI. White arrows indicate examples of gem structures in the red anti-SMN panel. (B) Graphical summary of averaged SMN gem counts in 300 GFP positive 3813 fibroblasts. Error bars represent ±SD. Statistical significance relating pM13 to pMU3 determined by values of p<0.05.

A frequently examined biomarker for SMN is a sub-cellular structure called gems. Gems are nuclear foci enriched in SMN and several SMA binding partners. Gems have proven a valuable tool to monitor the efficiency of SMN induction from a variety of therapeutic molecules, ranging from drugs to viral vectors. To determine whether pMU3 increased SMN gems, fibroblasts transfected with pMU3 plus a panel of negative controls were examined by indirect immunofluorescence. Transfected cells were readily detected due to the *GFP* expression from the plasmid backbone. In GFP-positive 3813 cells, pMU3 treated gems levels were significantly increased, resulting in nearly 60 gems per 100 nuclei and with approximately 30–40% of transfected cells expressing at least one to two gems (Figure 6A, 6B). Negative controls for fibroblast transfection and cellular reaction included plasmids lacking a viable tsRNA promoter sequence and empty pMU2 plasmid demonstrated therapeutically irrelevant increases in gem numbers (Figure 6A). Therefore we concluded that the ASO-tsRNA technology achieves the greatest biological impact relative to previous *SMN2* exon 7 *trans*-splicing theories in these well-characterized *in vitro* assays.

### pMU3-mediated *trans*-splicing increases SMN protein in SMA mice

**Figure 7:**
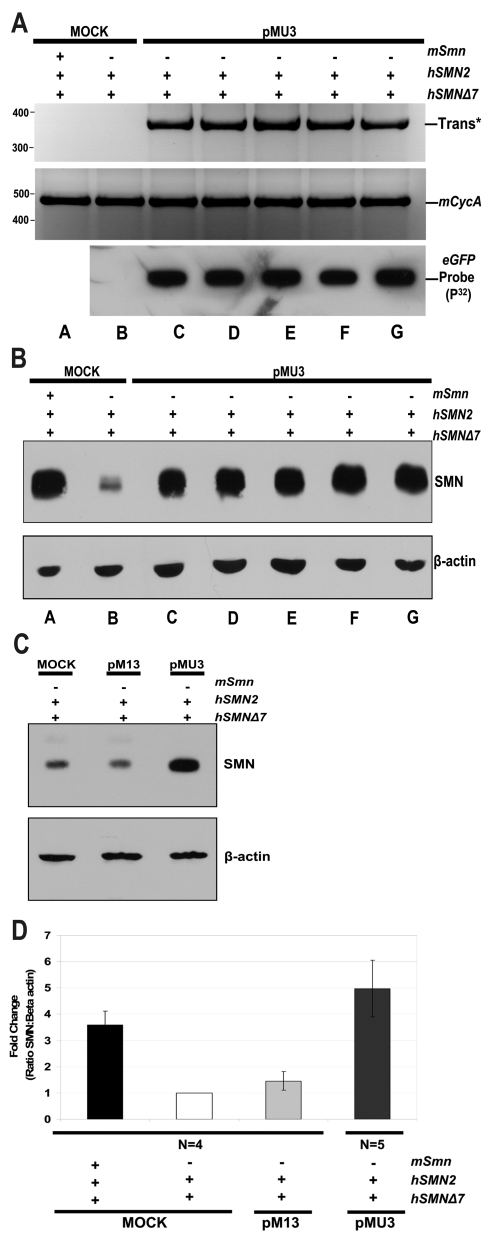
Intracerebroventricular 25 kDa PEI pMU3 transfections in SMA model mice increases SMN protein in the spinal cord. (A) Heterozygous (m*SMN* +/+, h*SMN* +/+, h*Δ7 SMN* cDNA +/+) (lane a) or homozygous (lanes b–g) (m*SMN* −/−, h*SMN* +/+, h*Δ7 SMN* cDNA +/+) neonatal mice (PND 0-1) were injected with pMU3 plasmid at 10 µg (1.14×10^12^ plasmid copies) with 25 kDa PEI over two ventricles (lanes c–g). Mock transfection mice are shown in lane a and b. Whole spinal cord homogenates were prepared for protein or RNA extraction 24 hours post injection. *In vivo* RT-PCR results for *trans*-spliced SMN are shown in the upper panel. Lower panel depicts controls for vector expression via northern blot of mock or treatment group spinal cord RNA loaded on nitrocellouse and developed with a radio-labeled GFP probe. (B) SMA mouse spinal cord protein was resolved on 10% SDS-PAGE. β-actin antibody panel serves as a normalization control. (C) Intra-ventricular transfections of pM13 in SMA model mice do not alter SMN levels in the spinal cord. Homozygous (m*SMN* −/−, h*SMN* +/+, h*Δ7 SMN* cDNA +/+) (lanes b,c) neonatal mice (PND 0-1) were injected with 10 µg of plasmid with 25 kDa PEI over both ventricles. Whole spinal cord homogenates were prepared for protein and resolved on 10% SDS-PAGE. β-actin antibody panel serves as a normalization control. Mock treatment is depicted in lane a. (D) Graphical summary of injection outcomes relative to SMN protein induction western blot in mice. Error bars represent ±s.d. HET, KO, pM13 (n = 4). pMU3 (n = 5).

To determine whether the cell-based analysis of pMU3 translated into comparable results *in vivo*, SMN proteins were examined in a previously described mouse model of SMA, referred to as *SMNΔ7* [24]. The SMA murine model expresses the genomic human *SMN2* gene and the human *SMNΔ7* cDNA. SMA neonatal mice were injected into both cerebral lateral ventricles with pMU3 or pM13 at 1.14×10^12^ plasmid copies using 25 kDa linear PEI and glucose. Negative control injections contained glucose, saline, and 25 kDa linear PEI to control for procedure and cytotoxicity. After 24 hours the spinal cord was dissected and used to generate total RNA and protein extracts. Utilizing *trans*-splicing specific RT-PCR primers we identified a positive *trans-SMN* mRNA product in the spinal cord (Figure 7A). Northern and southern blotting using a *GFP*-specific probe demonstrated that similar levels of plasmid were present in each of the treated SMA mice (lower panel Figure 7A, and data not shown, respectively). Western blots of disassociated whole spinal cord extracts from five individual pMU3-treated SMA pups were examined and shown to contain significantly elevated levels of SMN protein. pMU3-induced levels of SMN were significantly higher than untreated SMA mice and were comparable to SMN levels detected in homogenates from unaffected heterozygotes (Figure 7B). In contrast, the parental vector that lacked the ASO, pM13, did not result in significant levels of SMN induction (Figure 7C, 7D). Similarly, the ASO-alone vector failed to induce SMN levels *in vivo* (data not shown). Taken together, these results demonstrate that the pMU3 vector strategy that encodes two separate RNAs significantly increases *SMN trans*-splicing and provides the first demonstration of *SMN trans*-splicing *in vivo*.

## Discussion

This is the first demonstration of a technique designed to enhance *trans*-splicing efficiency by blocking downstream splice site selection. While the initial *trans*-splicing RNA alone showed considerable activity in cell-based models of SMA in terms of significantly increasing SMN levels and increasing snRNP activity [15], these results did not translate to the *in vivo* context. Our research highlights a key finding that low dose *trans*-splicing is enhanced by the co-expression of the ASO and resulted in detectable levels of SMN expression *in vivo*.

As a therapeutic approach, *trans*-splicing offers the advantage over gene replacement in that expression is intrinsically controlled by the endogenous promoter [7]. Consequently, temporal and spatial constraints on gene expression are retained. As *SMN* expression has previously been shown to be significantly down-regulated from embryogenesis to adulthood, this additional restraint may prove beneficial for long-term exposure to a *SMN trans*-splicing vector. Avenues of research directed to the reduction of tsRNA vector dosage would consequently inhibit the negative effects of off-target *trans*-splicing over greater amounts of time.

The genetic context of SMA represents an intriguing target for a number of therapeutic strategies, including *trans*-splicing. In the *SMN2* gene, the intrinsic quality of the exon 7 splice acceptor site is reduced due to the C/T transition. Therefore, the competition between *SMN2 cis*-splicing and *trans*-splicing is likely reduced, providing a potential advantage to *trans*-splicing in the *SMN* context compared to other alternatively regulated exons that retain fully functional splice sites. We propose *trans*-splicing is further enhanced relative to cis-splicing with the introduction of an inhibitory ASO. Since the tsRNA annealing domain hybridizes over the target exon, it is logical that this may promote exon 7 skipping. Therefore, the addition of the anti-sense likely blocks this escape pathway and further promotes trans-splicing. Unlike many ASOs that modify splicing, the ASOs used in this context are native RNA structures, not oligonucleotides with modified backbones. Therefore, the affects may be difficult to observe at steady-state levels when used as an ASO alone. However, given that this sequence overlaps the intron 7/exon 8 boundary, it was not surprising that the ASO recapitulated the increase in trans-splicing observed with the slow polymerase and intron 7-deleted construct.

In general, the field of *trans*-splicing therapeutics can benefit from the discovery of combined ASO-tiling and tsRNAs. ASO-tiling increases the potential of tsRNA by modulating *cis*-splicing signals within the target transcript. Additionally, in a novel application of ASO-In4^11^ and tsRNA^EX4^ co-delivery targeting a upstream *SMN* intron 3 exon 4 splicing context produced enhancement of basal *trans*-splicing. (Supplemental Figure S2) Studies here demonstrate the limitations of dual vector delivery in difficult fibroblast transfection conditions. Co-transfection dynamics hinder the potential of ASO/tsRNA pre-mRNA redirection and can be circumvented by sub-cloning of the single vector pMU3. The combination of ASO-tsRNA mechanism and a novel single vector platform produces a potent enhancement of basal *trans*-splicing. In the case of *trans*-splicing RNAs designs such as, 5′ or 3′ versions would require examination of beneficial upstream and downstream sequences to tile with ASO RNA.

The *in vivo* results demonstrate ASO-tsRNA represents a tractable therapeutic yet highlight the transient nature of plasmid transfections. The use of AAV vectors for the delivery of gene therapies would provide a substantial longevity to ASO-tsRNA expression and thus *SMN2* redirection. Future studies could examine the role of expression of the virus vectors and the SMA CNS. We conclude the combined effects of ASO-tsRNAs in a novel expression vector enhanced *trans*-splicing in the context of *SMN2* alternative *cis*-splicing. Applications of this biotechnology on a well characterized SMA model mouse demonstrate the promise of *in vivo* restoration of SMN via *trans*-splicing RNAs.

## Materials and Methods

### Plasmids and cloning

The pMU2-tsRNA^M13^ clone reported previously is now written as pM13 [15]. The *SMN1* and *SMN2* mini-genes (p*SMN1*, p*SMN2*) have been previously described [12]. Mini-gene p*SMN2* **^ΔIn7^** was created by using overlapping PCR primers which anneal the 5′ splice donor of exon 7 to the 3′ splice acceptor of exon 8. SMN In7 DEL FWD (5′- TCCTTAAATTAAGGAGAAATGCTGGCATAGAGC-3′) This PCR product was then gel purified and cloned into the pCI minigene using 5′-NheI and NotI-3′. The plasmid pAT7-*Rpb1*- (N792D) (R749H) expresses the mutant RNA polymerase [21]. The Antisense Oligonucleotides (ASO) was cloned into the previously reported pMU2 plasmid context driven by a U6 promoter [14]. Sequences were chosen with intron 6 dentoted as In6^X^ (with X indicated the sequential number) and intron 7 as In7^X^. Listed below are the binding domains for the ASO as read 5′ to 3′ in the *SMN* pre-mRNA **[** ***SMN*** **EXON 6]5′**- (In6^1^-5′-uucugaucauauuuuguugaauaaa-3′) (In6^2^-5′uuuguugaauaaaauaaguaaaaug) (In6^3^-5′auaaguaaaaugucuugugaaacaaa) (In6^4^-5′gucuugugaaacaaaaugcuuuuuaa) (In6^5^-5′aaugcuuuuuaacauccauauaaag) -**3′[** ***SMN*** **EXON7]5′**- (In7^1^-5′auaugggaauaaccuaggcauacugca) (In7^2^-5′cuaggcauacugcacuguacacucug) (In7^3^-5′cacuguacacucugacauaugaagug) (In7^4^-5′acauaugaagugcucuagucaa) (In7^5^-5′gcucuagucaaguuuaacuggugucca) (In7^6^-5′uuuaacugguguccacagaggacau) (In7^7^-5′acagaggacaugguuuaacugga) (In7^8^-5′gguuuaacuggaauucgucaagcc) (In7^9^-5′ggaauucgucaagccucugguucuaauuu) (In7^10^-5′ugguucuaauuucucauuugcagG) (In7^11^-5′ucucauuugcagGAAAUGCUGGCAUA) -**3′[** ***SMN*** **EXON8]** (In^712^-5′AAUGCUGGCAUAGAGCAGCACUAAA) (In7^13^-5′AUGACACCACUAAAGAAACGAUCA) (In7^14^-5′AAGAAACGAUCAGACAGAUCUGGAA) The pMU3 construct produces three gene products, tsRNA, eGFP protein and ASOs. ASO-In7^11^ was subcloned by PCR Platinum *Pfx* DNA Polymerase (Invitrogen) amplification according to the manufacture instructions of the U6 promoter and the antisense RNA and cloned by flanking restriction sites 5′-BlpI and AsiSI-3′(NEB). pMU3^KO^ is a substitution of the In7^11^ anti-sense RNA with a random sequence of RNA created by overlapping PCR and cloned using the same restrictions sites above. The pMU2-tsRNA^EX4^ intron 3 binding domain was amplified from HeLa genomic DNA using primers (Int3 Rev 5′- ATCAAGCTTGAGCTCAAAAAGAAAAAATATGCAGGTTTT-3′) and (Int3 Fw 5′-ATCACCGCGGTTCAATTTCTGGAAGCAGAGACTA-3′) and cloned into HindIII-SacII of pcDNA3/PTM2 replacing Int6 binding domain. Exons 4–7 were amplified from SMN1 cDNA clone using primers (Ex4 Fw 5′-TCAGATATCCTGCAGAGAATGAAAATGAAAGCCAAGTTT-3′) and (Ex7 Rev 5′- CATTCTCGAGGGGCCCGTCATAGCTGTTTCCTGCGACTCCTTAATTTAAGGAATGTGAGCA-3′) and cloned into EcoRV and XhoI sites of pMU2. The In4^11^ antisense fragment was amplified from pMU3 construct using BsrGI In7^11^ (Fwd 5′-AGCTGTACAAGTAAAGCGGCCGCGACTCTAGATC-3′) and MluI In4^11^ (Rev 5′- GTAACGCGTAAAAAATTCCTTATAGCCAGGTCTAAAATGGATCCGAAGACCACAAACAAG-3′) and was cloned to make pMU2-In4^11^, the antisense fragment into the BsrGI-MluI sites of pMU2 vector. Ligations were carried out using 10–15 units of T4 DNA Ligase (NEB) at 15°C for 24 hours and transformed in Subcloning Max Efficency DH5α. (Invitrogen) Sequencing of gel purified *trans*-splicing SMN M13 positive cDNAs produced from *in vivo* murine RT-PCR was performed using 1.5 ng of h*SMN* Exon 6 Forward primer and on a ABI 3730 DNA Analyzer and Applied Biosystems Big Dye Terminator system and read by Chromat Freeware.

### *In vitro* Transfections

The transfections were performed using linear PEI (Polysciences, Inc.) to pH 8.3 with 20 µL adjustments of HCl or NaOH. Stock linear 250 kDa PEI for HeLa in vitro transfections were snap frozen in liquid nitrogen and thawed 5 times at 37°C. Triple transfections in HeLas required “mean-low molecular weight PEI,” stock PEI was sonicated on ice at 30% total power for 30 seconds three times. Plasmids were transfected into HeLa or HEK293 cells (Coriell Cell Repositories) grown to 80% confluence before transfection. Plasmids were diluted in a 150 mM NaCl solution before adding filtered linear 7.5 mM 250 kDa PEI. Cells were incubated with plasmid overnight and replenished with fresh Dulbecco's Modified Eagle Media High Glucose 1× DMEM^+^ (D-glucose 4.5 g/L, +L-glutamine, 1% penicillin/streptomycin 10 000 units/mL, 3% fetal bovine serum-endotoxin free) and harvested 48 hours post-transfection. Controls include salmon sperm DNA (ssDNA) (Invitrogen) and normalized across co-transfection samples to greatest treatment plasmid DNA micrograms. SMA patient fibroblasts (GM3813) were transfected with Lipofectamine™2000 with LiCl (5M) centrifuge purified plasmid in serum free DMEM^−^ for 4 hours then washed via media and replaced with DMEM^+^ for 2 hours. Finally complete media DMEM^+^ was added back for the remaining incubation. Cells were harvested 24 hours later in PBS (137 mM NaCl, 2.7 mM KCl, 10 mM Na_2_HPO_4_, 2 mM KH_2_PO_4_) pH 7.5–8.0. ASO-tsRNA co-transfections used ssDNA to control for total volume of DNA. Post-transfection harvested cells were counted on a hemocytometer and use Trypan blue dye exclusion to determine viable cell counts per microliter.

### RT-PCR

48 hours post transfection or transduction, total RNA was harvested from cells using TRIzol Reagent (Invitrogen) according to the manufacturer's instruction. Total RNA concentration determined via Nano-drop OD260 and normalized before cDNA synthesis. RT-PCR was performed as previously described [14]. The *trans*-splicing product was amplified using a reverse primer M13 (5′-GTCATAGCTGTTTCCTGCGAC-3′) 5′ Cy3 labeled (564 nm emission max) (Intergrated DNA Technologies, Inc.) and a mini-gene specific primer, pCI-Fwd [12]. The SMN1 and 2 mini-gene transcripts were amplified using pCI-Fwd#1 and pCI-Rev [12]. The primers used in endogenous PCR amplifications were an *SMN* Exon 6 -Forward (5′-CCCCCACCACCTCCCATATGT-3′) and *SMN* Exon 8-Reverse (5′-AGTGGTGTCATTTAGTGCTGC-3′). The endogenous pMU2-tsRNA^EX4^ mediated *trans*-SMN product was amplified using primer Exon 3^+126^ (Fwd 5′-GAGAGGAGCAAAATCTGTCCGATCTAC-3′) and M13 Rev. The endogenous *trans*-spliced product is amplified by *SMN* Exon 6 (+7) Forward and M13 Reverse. Negative RT polymerase controls were included in protocol but omitted from final figures. Semi-quantitative determinations via Cy3 labeled *trans*-splicing products were imaged using laser Fuji-Imager FLA5000 at 550 nm excitation range. RT-PCRs were repeated in triplicate to confirm 2-tail t-test statistical significance of the increase in *trans-SMN* mRNA. To normalize within experiments, *trans-SMN* induction is determined as (*trans*-*SMN:GAPDH*) and between experiments levels were normalized to basal pM13 *trans*-splicing values (set to 1). *SMN trans*-splicing was measured using fold change over pM13 set to 1 as expressions of *trans*-splicing efficiency.

### Immunofluorescence microscopy

SMA patient fibroblasts (Coriell Cell Repositories, 3813 cells) harvested at sub-confluent levels were plated for transfections on UV irradiated glass coverslips. Prior to harvest, samples were washed twice in PBS pH 7.5. Fixation of cells was performed using cold acetone/methanol (50∶50) [14]. 1% BSA was used as a blocking medium for 1 hour then the samples were washed in PBS pH 7.0. Primary antibody 4B7 mouse anti-SMN was diluted 1∶500 in PBS. Secondary antibody (goat anti-mouse Texas Red-594: Jackson Immuno-Research Laboratories) were used according to manufacturer's instructions. Cells were washed with PBS and nuclei stained with DAPI [25]. Microscope images were captured on Nikon Eclipse E1000 using Meta-Morph software. Control exposures determining levels of background for the secondary in the absence of primary antibody set basal limits for SMN detection. These controls are performed during each experimental round to control for intra-assay perturbations.

### Western blot

HeLa cell pellets were prepared in RSB100 lysis buffer (20 mM Tris-HCl pH 7.4 100 mM NaCl, 25 mM MgCl_2_, Triton ×100 0.5%) and centrifuged at 10,000 g for 5 min at 4°C. Samples were sonicated and boiled in loading dye then resolved in a 10% SDS-PAGE 20 cm×10 cm BioRad Protean xi Glass plates using Protean 2000. Resolved proteins were then transferred to PVDF (Immoblion) at 200 mAmps for 400 minutes in Towbins (24 mM Tris base, 192 mM glycine, 20% methanol (v/v)). Western blots were blocked overnight in 5% Casein and Tris-buffered Saline (TBS^T^) (90 mM Tris-HCl, 10 mM KCl, 547 mM NaCl, 2% Tween (v/v) (Acros)) pH 7.5. Primary patient fibroblast western blots were probed with 1∶100 dilution of mouse anti-SMN monoclonal antibody, 4B7 [25], and visualized with a HRP-conjugated secondary mouse antibody. Murine western blots were probed with 1∶1000 dilution of mouse anti-SMN (BD Biosciences) and were produced using 1∶1 mixture of Peirce West Pico reagent. Cross reaction with endogenous mouse heavy and light chain antibody Mouse TrueBlot™ ULTRA HRP anti-mouse IgG (eBioscience) was used at 1∶10 000 in 5% skim milk. Images were captured and quantitated using a Fuji Imager LAS 3000 at 75% of total resolution 10–30 second exposure and Multi-Gauge V2.3 system. To control for loading error the westerns were then stripped using H_2_O_2_ for 15–20 minutes at room temperature and re-probed with anti-β-actin rabbit and anti-rabbit HRP. Western blots were repeated in quadruplicate to confirm 2-tail t-test statistical significance of the increase in SMN protein. To normalize within experiments, SMN induction is determined as (SMN:β-actin) and between experiments SMN levels is normalized to GM3813 values (triplicate mean set to 1). SMN protein induction was measured using pIn7^11^ ASO as baseline, and fold change over pM13 alone (triplicate mean set to 1) as expressions of *trans*-splicing efficiency.

### *In vitro* snRNP assembly reactions

Cloned U1 snRNA (ΔSm) cDNA has the Smith core site (5′-AUUUGUGG-3′) sequence deleted to inhibit non-specific products [26]. The U1 snRNA (WT and ΔSm) was in vitro transcribed using the Maxi-prep (Invitrogen) plasmid pT7 cleaved with *Eco*RI for 3 hours and cleaned up with PCR columns (Invitrogen). U11^ATAC^ snRNA was synthesized with mature post-transcriptional modification sequences and cloned into pGA4 (GENEART) flanked by T7 sequences. Transcriptionally competent snRNA^ATAC^ templates were created with primers and high fidelity PCR amplification (Roche). U11FWD #1 (5′-GATCGATCTAATACGACTCACTATAGGGAGAATTCAAAAAGGCTTCTGTCGTGAG-3′) U11REV #1 (GATCGATCGGATCTAAATCCACTGTGATATCTTCTCAAAGGGCGCCGGGACCAA-3′) The T7-U11^ATAC^ PCR product was PCR purified and used in subsequent transcription reactions as a template. snRNA transcription reactions: 10 units of T7 RNA polymerase HC (Ambion), ARCA-Methyl cap (Ambion) and rNTPs supplemented with P^32^ labeled rUTP was incubated for 30 minutes at 30°C. Cells were harvested and lysed in on ice via fresh Reconstitution Buffer (20 mM Hepes-KOH pH 7.9, 50 mM KCl, 5 mM MgCl2, 0.2 mM EDTA, 0.01% Triton ×100). Cytoplasmic and nuclear fractioning was accomplished by centrifugation at 1000 rpm for 15 minutes at 4°C. The S100 fraction was removed and (7×) EDTA free protease inhibitor cocktail (Roche) added to preserve the SMN complex. Assembly of the Sm cores occurred for 30 minutes at 30°C using 20U of RNAsin (Promega) added to 50 µg total of S100 extract at final concentration of 2.5 mM ATP, 1.0 µg Yeast tRNA, U1 snRNA (100,000 cpm). For the general assembly in the U11^ATAC^snRNA 75 µg total extract was required for 60 minutes at 30°C. Following assembly, 350 uL of DEPC-RSB^200^ buffer was used in the initial immunoprecipation (IP) steps via primary bead fractions Protein G Plus/Protein A Agarose suspension with PBS (Oncogene) pre-blocked with 25 µg of whole HeLa cell extracts. Bead fractions are pre-incubated with 1 uL anti-Mouse Y12 mono-clonal antibody (Lab Vision) per 50 µL stock slurry volume. Radio-IPs were processed at 4°C to reduce signal loss and washed at 25°C. UsnRNP and beads were denatured in (5×) formamide loading dye and run on an 8% acrylamide TBE-Urea gel (45 mM Tris-borate, 1 mM EDTA, 7M Urea). Quantitative measurements of snRNP products were derived after a 30–45 minutes exposure on a phospho-screen (Kodak). Bands were imaged using Fuji-Imager FLA5000 and Image Reader FLA5000 V2.0 software (IP^−S^ mode) normalized to background. The Fuji-imager results were correlated to excised bands via scintillation counter output averaged over three rounds of counts per minute. Within experiments the capacity to assemble snRNPs is determined by the ratio of U1^WT^∶U1^KO^. Between experiments, comparisons were determined by normalization to GM3813 values (set to 1) and above the assembly baseline of ASO pIn7^11^ transfections. GM3813/3814 fibroblasts were included in the experiment up to the fourth passage post cryo-preservation rescue to avoid artificial selection of SMN positive populations.

### Intracerebroventricular injection of SMA mice

SMA mouse were genotyped PND 0 via tail clip. Frozen tails were prepared using a binary HOTShot method: 90°C for 10 minutes alkaline lysis solution (25 mM NaOH 0.2 mM disodium EDTA) and Trizma base neutralizing solution (40 mM Tris-HCl). 1.5–2.0 µL input tail solution was added to PCR reactions containing previously described, yet renamed for clarity, primer sets *mSMN* FWD (5′-GCAGCTGTGCTCGACGTTGTC-3′), *mSMN* REV (5′-TAAGAAAGCCTCAATGTGCTCAAG), h*SMN2* FWD (5′-GCGATAGAGTGAGACTCCATCT-3′), h*SMN2* REV (5′-GACATAGAGGTCTGATCTTTAGCT-3′) [27]. PND 2 neonates were immobilized via cryo-anesthesia and injected using µL calibrated sterilized glass micropipette 0.25 mm lateral to the sagittal suture and 0.50–0.75 mm rostral to the neonatal coronary suture. The needles were inserted perpendicular to the skull surface using a fiber-optic light (Boyce Scientific Inc.) to illuminate pertinent anatomical structures. Needles were removed after 15 seconds of discontinuation of plunger movement to prevent backflow. Mice recovered in 5–10 minutes in a warmed container until movement and general response was restored. Injection stock solutions contained and final volumes include: D-(+)-glucose 20% (w/v) (1 µL) (Sigma), trypan blue (0.4%) saline (1 µL) (Sigma), plasmid (≈5 µg/µL) (2 µL), 25 kDa linear PEI homopolymer (150 mM) (1 µL) (Polysciences, Inc.). *In vivo* plasmids prepared with endo-toxin free Maxi-prep (QIAgen) were analyzed for purity and superhelicity in 4% agarose gels and NanoDrop quantifications. Control mock in vivo transfections contain glucose, trypan blue saline, 25 kDa PEI and do not contain plasmid. *In vivo* western and northern blot was performed by dissecting the CNS with microsurgical technique via Roboz RS-5600 (Roboz Surgical Instrument Co., Inc.) and gross disassociation performed by passing tissue re-suspended in PBS through a 18-gauge needle and aliquot into individual tubes, then snap-frozen in liquid nitrogen. Total spinal cord RNA was collected by TRIzol (Invitrogen) and the final pellet resuspended in 30 µL of PrepSolution (40 mM PIPES pH 6.8, 1 mM EDTA, 250 mM NaCl, 80% (v/v) deionized formamide). The RNA was blotted by vacuum with BioDot SF (BioRad) seeding on 9×12 cm Zeta probe membranes (BioRad). GFP probes were created by Klenow fragment synthesis with Easytides dATP creating 10^8^ cpm/µg product used at a final concentration of 10^6^ cpm/mL in the hybridization buffer (500 mM Na2PO4 pH 7.2, 1% SDS, 1 mM EDTA). *In vivo* murine *trans*-splicing RT-PCR was performed with the previously reported “human-specific” Exon 4 35–55 forward (Hua et al. 2008) combined with the standard M13 reverse primer.

### Statistical analysis

Student t-tests were utilized to gauge significance between two results via 2-tailed regression analysis. Levels of significance were set to p≤0.05 for a single treatment regiment and repeated in triplicate to determine mean and ±standard deviation values. Statistical values are represented as the inset graphs and error bars indicate ±s.d. for the averages. The data collected was normalized internally to 1 using a base line controls indicated in the above method sections. This approach is justified by the number of mice compared, involved in treatment versus mock, and relative fold change in the overall protein expression post-treatments using SMN^KO^ mouse mock as basal.

## Supporting Information

Figure S1 Summary of SMN2 trans-splicing and intron 7 ASO-tiling screen demonstrates specificity of ASO-In711 effect. (A) Graphical depiction of intron 7 ASO targets. ASO sequences are identified at the beginning of the individual string, written 5′-3′. Capitalized sequence indicates exon 8 sequences with the 3′ splice site defined in red. The bars located directly below illustrate the effects on the trans-splicing product by the respective colors. Black lines indicate neutral effects, red lines indicate negative influences, and green lines represent increased trans-splicing. (B) Summary of even numbered intron 7 ASO screen demonstrates no effect. HeLa cells were triple-transfected with plasmids expressing pM13 1.0 µg, a minigene SMN2 (lanes a–h) 1.25 µg and ASO pIn72-pIn712 (lanes c–h) 1.0 µg and RNA harvested at 48 hrs. Reverse transcriptase PCR gel is displayed with GAPDH normalization control. pIn71 negative control serves to normalized between ASO experiments. (C) Summary of odd numbered intron 7 ASO screen identifies pIn711 ASO as a potent enhancer of trans-splicing. HeLa cells were triple-transfected with plasmids expressing pM13 1.0 µg, a minigene pSMN2 (lanes a–d) 1.25 µg and ASO pIn71-5, pIn77-11 (lanes b–d) 1.0 µg for blots respectively and RNA harvested at 48 hrs. Reverse transcriptase PCR gel is displayed with GAPDH normalization control. pM13 controls for basal trans-splicing. (D) HeLa cells were triple-transfected with plasmids expressing a minigene SMN2 (lanes a–d) 1.25 µg, pM13 (lanes b–e) 1.0 µg, a negative control scrambled ASO-tsRNA vector pMU3KO (lane a) 1.0 µg and ASO-tsRNA DNA recombination control transfection with pM13 and pIn711 (lane e) 1.0 µg minus pSMN2. RNA harvested at 48 hrs. Reverse transcriptase PCR gel is displayed with GAPDH normalization control. pM13 controls for basal trans-splicing (lane c).
(2.16 MB TIF)

Figure S2 The ASO-tsRNA mechanism can be applied to heterologous contexts. SMA patient fibroblasts co-transfected with a trans-splicing RNA that targets SMN intron 3 containing SMN exons 4–7 written as “pMU2-tsRNAEX4” (lanes b–f) (0.25 µg) with increasing concentrations of enhancing ASO directed toward the intron 4/exon 5 splice site “pMU2-In411” (c–f) (0.25 µg, 0.50 µg, 0.75 µg, 1.0 µg) produced increased trans-splicing. Reverse transcriptase PCR gel is displayed with GAPDH normalization control. Lane a represents negative control omitting RT polymerase, lane b is base line trans-splicing for pMU2-tsRNAEX4.
(1.65 MB DOC)
