# Supplementary figures and images for: Development of a Single Vector System that Enhances Trans-Splicing of SMN2 Transcripts
Source: PLoS One. 2008 Oct 22;3(10):e3468. doi: 10.1371/journal.pone.0003468 (PMC2565107; doi:10.1371/journal.pone.0003468)

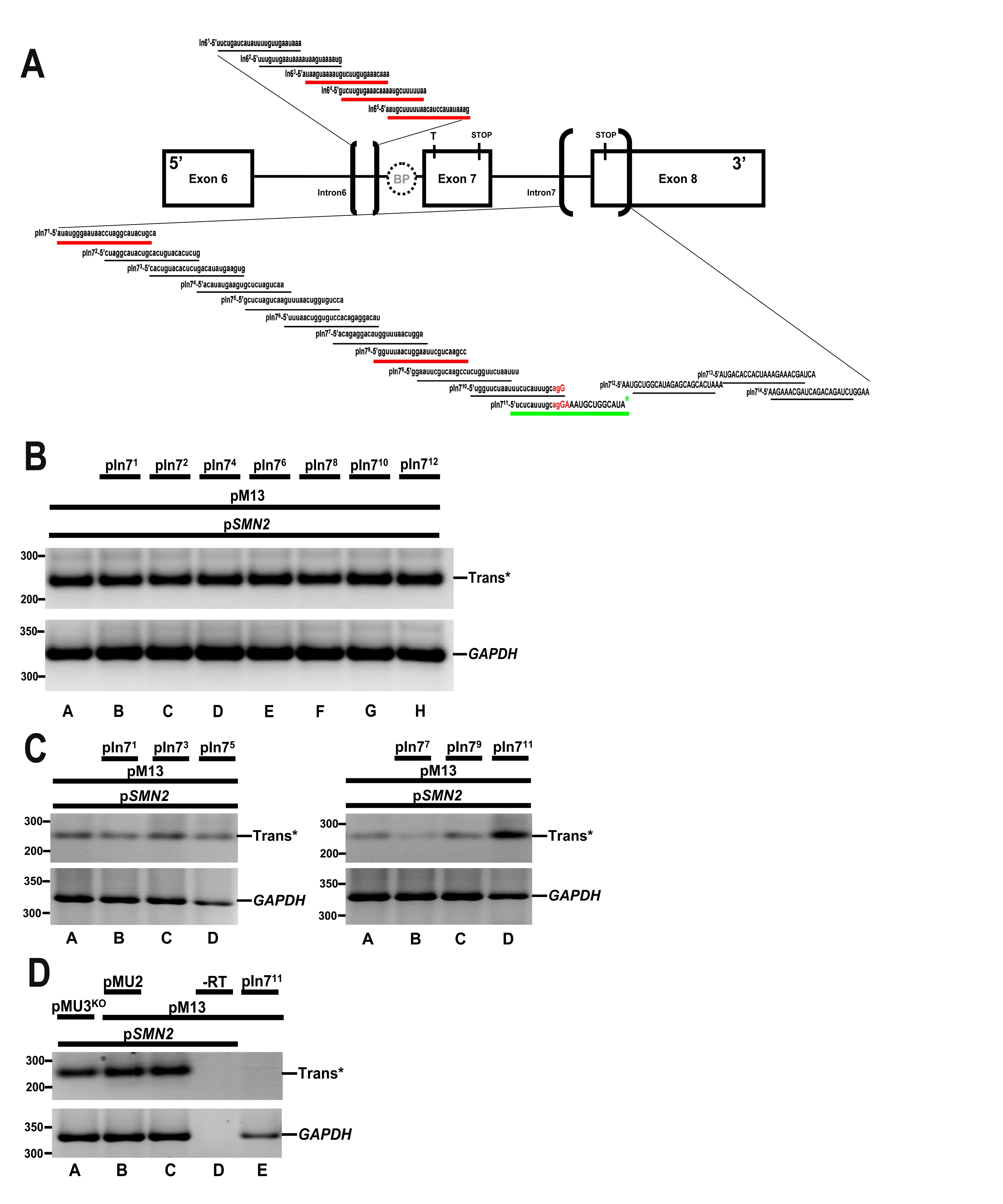

Supplement: Figure S1 — Summary of SMN2 trans-splicing and intron 7 ASO-tiling screen demonstrates specificity of ASO-In711 effect. (A) Graphical depiction of intron 7 ASO targets. ASO sequences are identified at the beginning of the individual string, written 5′-3′. Capitalized sequence indicates exon 8 sequences with the 3′ splice site defined in red. The bars located directly below illustrate the effects on the trans-splicing product by the respective colors. Black lines indicate neutral effects, red lines indicate negative influences, and green lines represent increased trans-splicing. (B) Summary of even numbered intron 7 ASO screen demonstrates no effect. HeLa cells were triple-transfected with plasmids expressing pM13 1.0 µg, a minigene SMN2 (lanes a–h) 1.25 µg and ASO pIn72-pIn712 (lanes c–h) 1.0 µg and RNA harvested at 48 hrs. Reverse transcriptase PCR gel is displayed with GAPDH normalization control. pIn71 negative control serves to normalized between ASO experiments. (C) Summary of odd numbered intron 7 ASO screen identifies pIn711 ASO as a potent enhancer of trans-splicing. HeLa cells were triple-transfected with plasmids expressing pM13 1.0 µg, a minigene pSMN2 (lanes a–d) 1.25 µg and ASO pIn71-5, pIn77-11 (lanes b–d) 1.0 µg for blots respectively and RNA harvested at 48 hrs. Reverse transcriptase PCR gel is displayed with GAPDH normalization control. pM13 controls for basal trans-splicing. (D) HeLa cells were triple-transfected with plasmids expressing a minigene SMN2 (lanes a–d) 1.25 µg, pM13 (lanes b–e) 1.0 µg, a negative control scrambled ASO-tsRNA vector pMU3KO (lane a) 1.0 µg and ASO-tsRNA DNA recombination control transfection with pM13 and pIn711 (lane e) 1.0 µg minus pSMN2. RNA harvested at 48 hrs. Reverse transcriptase PCR gel is displayed with GAPDH normalization control. pM13 controls for basal trans-splicing (lane c). (2.16 MB TIF) [file pone.0003468.s001.tif]

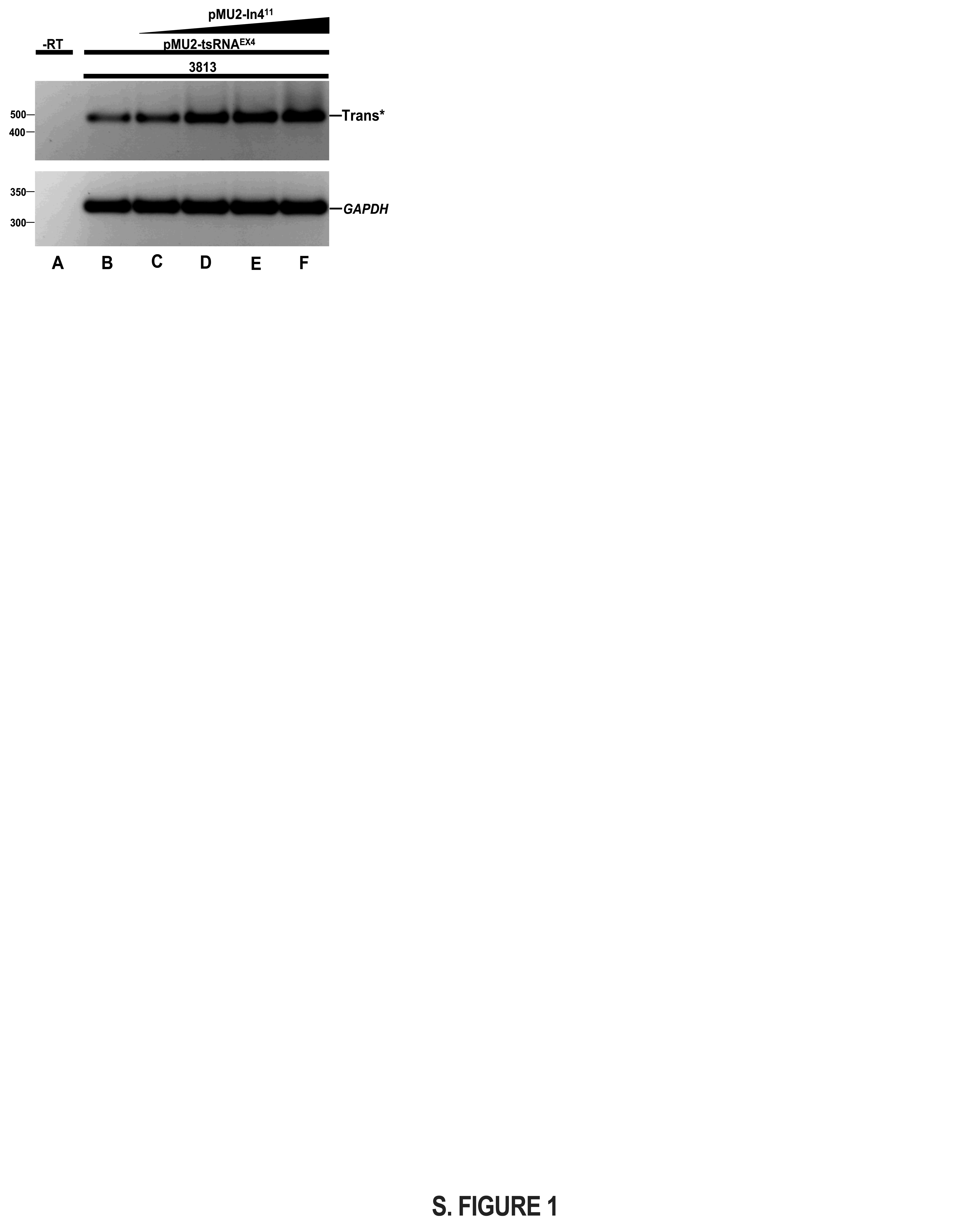

Supplement: Figure S2 — The ASO-tsRNA mechanism can be applied to heterologous contexts. SMA patient fibroblasts co-transfected with a trans-splicing RNA that targets SMN intron 3 containing SMN exons 4–7 written as “pMU2-tsRNAEX4” (lanes b–f) (0.25 µg) with increasing concentrations of enhancing ASO directed toward the intron 4/exon 5 splice site “pMU2-In411” (c–f) (0.25 µg, 0.50 µg, 0.75 µg, 1.0 µg) produced increased trans-splicing. Reverse transcriptase PCR gel is displayed with GAPDH normalization control. Lane a represents negative control omitting RT polymerase, lane b is base line trans-splicing for pMU2-tsRNAEX4. (1.65 MB DOC) [file pone.0003468.s002.doc]
